# Pulmonary surveillance in pediatric hematopoietic stem cell transplant: A multinational multidisciplinary survey

**DOI:** 10.1002/cnr2.1501

**Published:** 2021-07-28

**Authors:** Shivanthan Shanthikumar, William A. Gower, Matthew Abts, Deborah R. Liptzin, Elizabeth K. Fiorino, Anne Stone, Saumini Srinivasan, Timothy J. Vece, Nour Akil, Theresa Cole, Kenneth R. Cooke, Samuel B. Goldfarb

**Affiliations:** ^1^ Respiratory and Sleep Medicine Royal Children's Hospital Parkville Victoria Australia; ^2^ Respiratory Diseases Murdoch Children's Research Institute Parkville Victoria Australia; ^3^ Department of Paediatrics University of Melbourne Parkville Victoria Australia; ^4^ Division of Pediatric Pulmonology, Department of Pediatrics University of North Carolina Chapel Hill North Carolina USA; ^5^ Seattle Children's Hospital Seattle Washington USA; ^6^ Pediatric Pulmonary and Sleep Medicine University of Washington Seattle Washington USA; ^7^ Department of Pediatrics University of Colorado School of Medicine Aurora Colorado USA; ^8^ Division of Pulmonology, Allergy, and Immunology, Department of Pediatrics Weill Cornell Medicine New York New York USA; ^9^ Division of Pulmonology, Department of Pediatrics Oregon Health & Science University Portland Oregon USA; ^10^ Division of Pulmonology, Department of Pediatrics University of Tennessee Health Science Center Memphis Tennessee USA; ^11^ Allergy and Immunology Royal Children's Hospital Parkville Victoria Australia; ^12^ Department of Oncology Sidney Kimmel Comprehensive Cancer Center, Johns Hopkins University School of Medicine Baltimore Maryland USA; ^13^ Division of Pulmonary and Sleep Medicine, Department of Pediatrics, School of Medicine University of Minnesota Minneapolis Minnesota USA

**Keywords:** diagnostic screening programs, pediatrics, respiratory tract diseases, stem cell transplantation

## Abstract

**Background:**

Hematopoietic Stem Cell Transplant (HSCT) is an established treatment for malignant and non‐malignant conditions and pulmonary disease is a leading cause of late term morbidity and mortality. Accurate and early detection of pulmonary complications is a critical step in improving long term outcomes. Existing guidelines for surveillance of pulmonary complications post‐HSCT contain conflicting recommendations.

**Aim:**

To determine the breadth of current practice in monitoring for pulmonary complications of pediatric HSCT.

**Methods:**

An institutional review board approved, online, anonymous multiple‐choice survey was distributed to HSCT and pulmonary physicians from the United States of America and Australasia using the REDcap platform. The survey was developed by members of the American Thoracic Society Working Group on Complications of Childhood Cancer, and was designed to assess patient management and service design.

**Results:**

A total of 40 (34.8%) responses were received. The majority (62.5%) were pulmonologists, and 82.5% were from the United States of America. In all, 67.5% reported having a protocol for monitoring pulmonary complications and 50.0% reported adhering “well” or “very well” to protocols. Pulmonary function tests (PFTs) most commonly involved spirometry and diffusion capacity for carbon monoxide. The frequency of PFTs varied depending on time post‐HSCT and presence of complications. In all, 55.0% reported a set threshold for a clinically significant change in PFT.

**Conclusions:**

These results illustrate current variation in surveillance for pulmonary complications of pediatric HSCT. The results of this survey will inform development of future guidelines for monitoring of pulmonary complications after pediatric HSCT.

## 1 INTRODUCTION

Hematopoietic Stem Cell Transplant (HSCT) is an established treatment for both malignant and non‐malignant conditions.^1^, ^2^ The rates of HSCT are increasing, and currently more than 50 000 ^3^ are performed annually worldwide including approximately 1600 HSCTs in the pediatric age group.^4^ Survival following HSCT has improved, with reduced relapse‐ and non‐relapse‐related mortality over time,^5^, ^6^, ^7^ and survival expected well into adulthood. The combination of increased HSCT and improved survival means there are increasing numbers of HSCT survivors who need surveillance for late effects of HSCT.^2^, ^7^ A recent study found that adults who underwent HSCT in childhood had a 14.4‐fold increased risk for death compared with the general population.^7^ Pulmonary complications comprise a leading cause of non‐relapse morbidity and mortality after HSCT.^7^, ^8^

Among children, there are multiple late pulmonary complications of HSCT, including infection, graft vs. host disease (GVHD), pulmonary vascular disease, interstitial fibrosis and others.^9^, ^10^, ^11^ Two categories of chronic lung disease, defined based on pulmonary function testing (PFT), are observed in the months and years following allogeneic HSCT: obstructive and restrictive lung disease.^10^, ^12^, ^13^, ^14^, ^15^ Obstructive lung disease, most commonly manifests as bronchiolitis obliterans syndrome (BOS) which is the most recognized form of chronic lung disease after HSCT. BOS is characterized by progressive, narrowing and destruction of small airways along with fixed airflow obstruction and is associated with increased morbidity and mortality.^8^ The incidence of BOS after HSCT is 4.8%–6.5%^16^, ^17^, ^18^ with a median time to development of 12.2 months.^19^ Restrictive lung disease occurs in a smaller proportion of patients with a median time to diagnosis of 11 months post‐HSCT.^20^, ^21^ This pattern is less specific for a single diagnosis, and is often multifactorial, including individuals with previous chest wall/thoracic surgery, radiation therapy, and deconditioning with neuromuscular weakness, as well as patients who develop acute lung injury (e.g. idiopathic pneumonia syndrome) and interstitial lung disease.

The clinical presentation of pulmonary complications of HSCT is often non‐specific, and investigations such as PFTs, chest imaging, bronchoscopy with bronchoalveolar lavage, and lung biopsy are employed to elucidate the exact cause. Pulmonary complications can often be detected non‐invasively, without ionizing radiation, and at relatively low cost by PFTs prior to the onset of clinical symptoms. This is important because early treatment has been shown to have profound implications for long term pulmonary health.^22^, ^23^, ^24^ Two pediatric cohorts have examined the efficacy of monitoring pulmonary function testing longitudinally and revealed that compared to clinical signs and symptoms, screening PFTs led to earlier identification of pulmonary complications.^25^, ^26^ Screening for post‐HSCT pulmonary complications with PFTs to facilitate early detection and treatment is generally considered standard of care.

Despite consensus that screening for post‐HSCT pulmonary complications should occur, existing guidelines include conflicting recommendations regarding which tests should be performed, the frequency of testing and omit recommendations for patients unable to complete PFTs.^1^, ^2^, ^27^ For example the Children's Oncology Group^2^ recommend annual screening with spirometry and diffusion capacity for carbon monoxide (D_L_CO) only, whereas a multi‐society guideline^27^ does not specify which tests to use and recommends screening at 6 months post HSCT, 12 months, and then annually with increased frequency in those with graft vs host disease (GVHD). A guideline^1^ aimed at patients who underwent HSCT for hemoglobinopathy recommends screening at 3, 6, and 12 months post HSCT and then annually for a further 2 years, with screening involving spirometry, plethysmography and D_L_CO. In addition, current guidelines do not include tests such as multiple breath washout (MBW) or impulse oscillometry, which may be more sensitive measures of peripheral airway function (where BOS occurs) and are feasible in young children.^28^, ^29^ There is also little guidance for how other investigations, such as CT, bronchoscopy and bronchoalveolar lavage and biopsy should be used. Furthermore, many of these modalities, as well as access to pediatric pulmonology expertise, are not routinely available at all centers worldwide.^30^ Considering that early childhood is a critical period for lung development with long‐lasting effects into adulthood,^31^ optimizing pulmonary health in children post‐HSCT is crucial. An opportunity therefore exists to improve screening for pulmonary complications of childhood HSCT at the international level. This was one of the key messages from a recent National Institutes of Health workshop regarding pulmonary complications of HSCT.^32^ The workshop specifically called for standardization of lung function testing in this population, including the use of novel methods in the preschool age group.

The current project was established to better understand current multinational clinical practice. It was hypothesized that given the conflicting recommendations in guidelines, that there would be variation in practice between HSCT centers. In addition to inter‐center variation, it was also suspected that HSCT physicians and pediatric pulmonologists would differ in their approach to post HSCT patients. As such, the aim was to survey both HSCT physicians and pediatric pulmonologists regarding their current practice of screening for and diagnosing pulmonary complications of HSCT. Some of the results of this study has been previously reported in the form of an abstract.^33^

## 2 METHODS

### 2.1 Study population

An electronic web‐based survey of HSCT physicians and pediatric pulmonologists was conducted. Institutional review board approval was obtained from the Royal Children's Hospital, Melbourne, Australia (HREC number 2019.230). Using publicly available data,^34^, ^35^ a list of HSCT centers in the United States of America (USA) and Australasia (Australia and New Zealand) was created. These two regions were selected for survey as members of the study team reside and practice in these countries and were therefore most familiar with centers and practices therein. In order to survey a broad range of clinical practice the survey, one HSCT physician and one pediatric pulmonologist from each center were invited via email to complete an electronic survey. The department head or a clinician known to be interested in the area were chosen to receive the initial invitation. The recipient could forward the survey link to another more suitable member of their team as necessary. The survey was conducted in REDCap (Research Electronic Data Capture, Vanderbilt University), a web‐based application serving as a data capture instrument and repository. In the event no response was received, weekly reminders were automatically sent for 3 weeks. Participants provided informed consent as part of the survey, and responses were confidential and recorded in an encrypted database.

### 2.2 Study instrument

The survey instrument (see Appendix A) was developed by a core group of members of the American Thoracic Society Working Group on Complications of Childhood Cancer. The survey was then tested by separate members of the working group who provided feedback for improvement. The core group then revised the survey, and it was again tested by separate members of the working group prior to distribution. The survey consisted of 37 questions divided among six domains; general demographics, standard follow up, pulmonary function testing, imaging, access to pediatric pulmonology, and invasive diagnostic testing.

### 2.3 Analysis

As data was non‐normally distributed, correlations were tested using the Mann Whitney test, with *p*‐values <.05 considered significant. All statistical analyses were performed using GraphPad Prism, version 6.01 (GraphPad Software Inc., La Jolla, California).

## 3 RESULTS

The survey was distributed to 115 participants from 68 different HSCT centres on October 14, 2019. The survey was distributed to both a HSCT physician and pediatric pulmonologist at 47 centres, a HSCT physician only at 12 centres, and a pediatric pulmonologist only at 9 centers. A total of 40 (34.8%) responses were received, and all of them were complete and included in the analysis (Table 1).

**TABLE 1:** Summary of survey distribution and responses

	Distributed (*n*)	Responses
**United States of America**		
HSCT	52	15
Pediatric pulmonologist	49	18
Sub‐total	101	33
**Australasia**		
HSCT	7	3
Pediatric pulmonologist	7	4
Sub‐Total	14	7
**Overall**	**115**	**40**

### 3.1 General demographics

**FIGURE 1:**
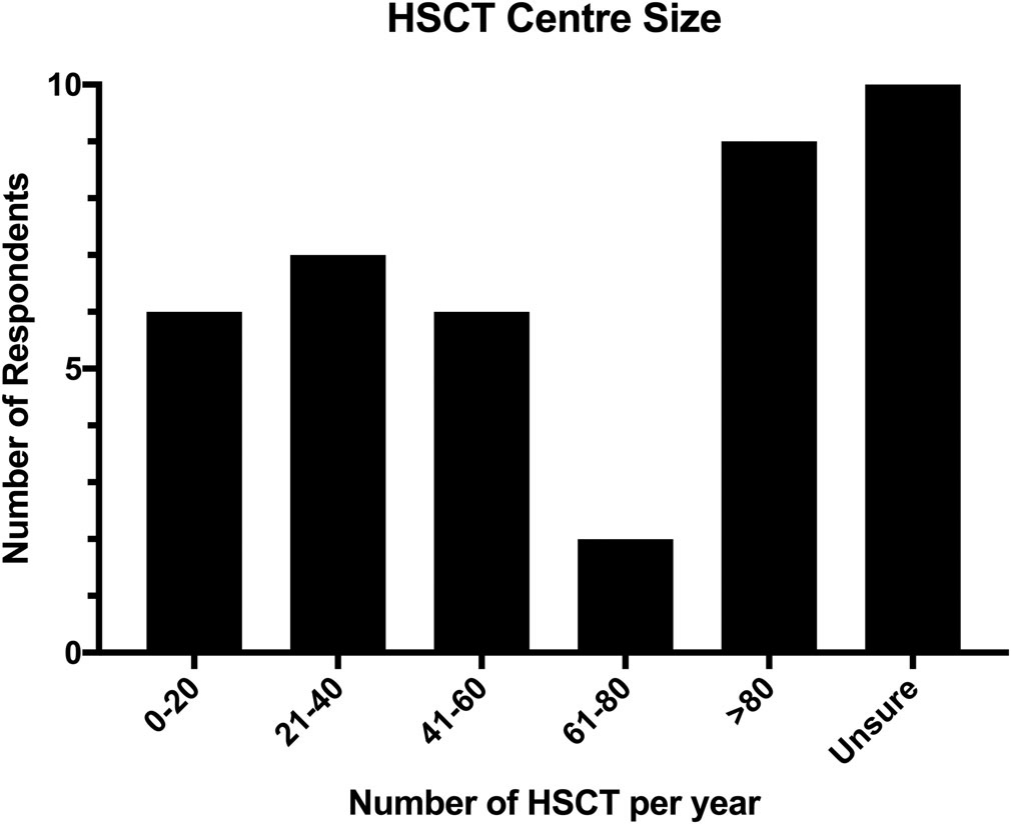
Average number of HSCT performed per year at respondents centers

Of the responses, 33/40 (82.5%) were from USA, and 25/40 (62.5%) were pediatric pulmonologists. All respondents worked at centers that performed both allogenic and autologous HSCT. All respondents worked at centers that performed HSCT for oncological indications; 36/40 (90%) also performed them for immune deficiency/ immune dysregulation and 34/40 (80%) for hemoglobinopathy. There was a large distribution in the average number of transplants performed per year which is summarized in Figure 1.

### 3.2 Standard follow up

The majority of respondents (27/40, 67.5%) reported that a protocol for assessing pulmonary complications of pediatric HSCT existed at their institution, however 20% (8/40) reported no protocol existed and the remainder were unsure about the existence of a protocol at their center. There was no significant difference in responses to this question between pulmonologists and HSCT physicians (64% vs. 73.3%, *p* = .68). Half of respondents felt their center adhered “well” or “very well” to their protocol (20/40, 50%), however 25% (10/40) reported “poor” or “average” adherence. Again, there was no difference in responses between pulmonologists and HSCT physicians (*p* = .56). For 17/40 (42.5%) the type of HSCT (autologous vs. allogeneic) affected the surveillance protocol, however for 15/40 (37.5%) it did not. More than half (23/40, 57.5%) of respondents reported that the indication for HSCT did not affect surveillance protocol.

### 3.3 Pulmonary function testing

**FIGURE 2:**
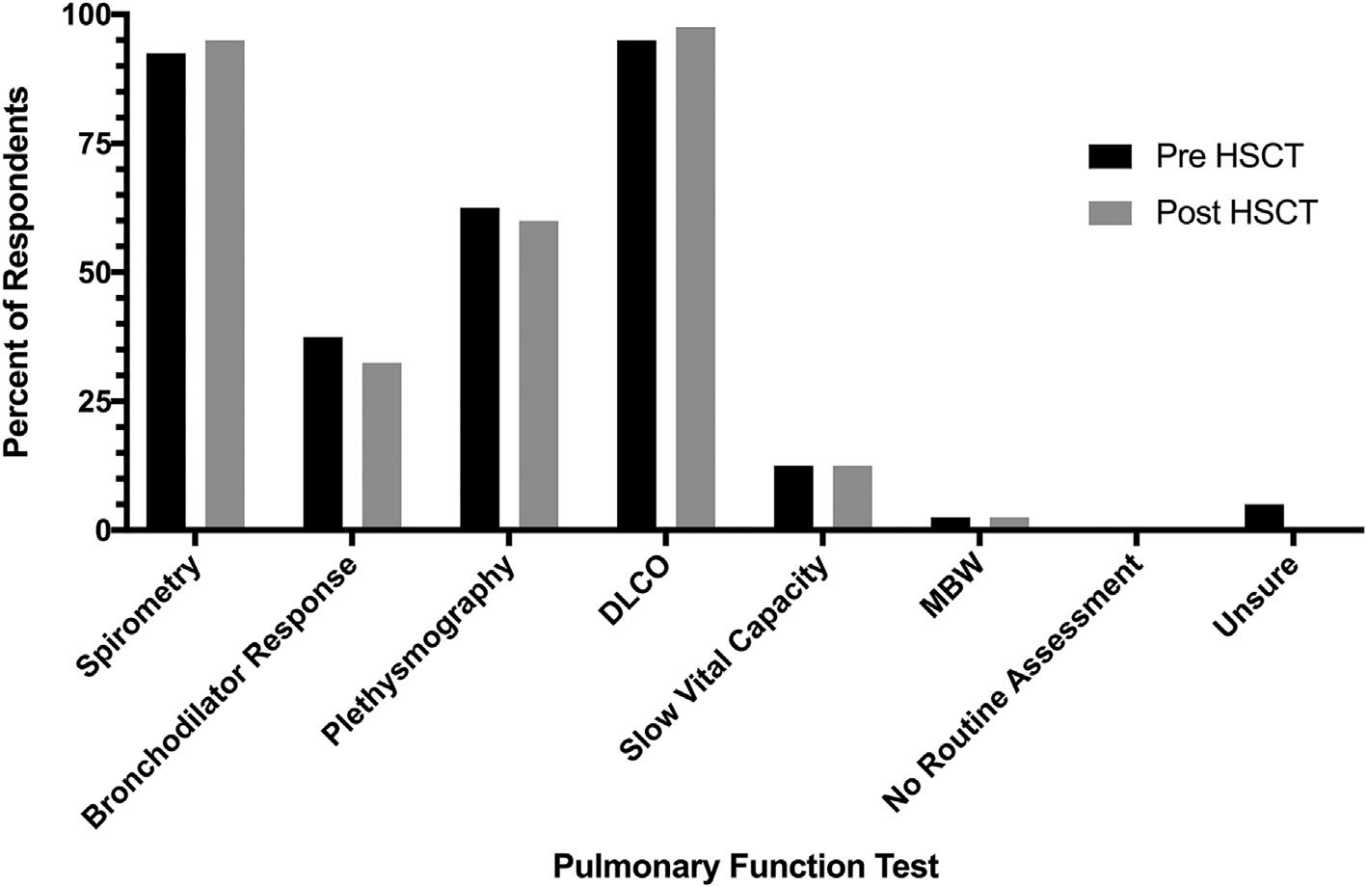
Summary of Pulmonary Function Tests performed before and after HSCT; DLCO (Diffusing capacity of the lungs for carbon monoxide)

The PFTs reported to be routinely performed pre‐ and post‐HSCT are summarized in Figure 2. Diffusing capacity of the lungs for carbon monoxide (D_L_CO) (38/40, 95%) and spirometry (37/40, 92.5%) were the most commonly performed PFTs prior to HSCT. The majority (38/40, 95%) of respondents reported routine post‐HSCT PFTs, and again, D_L_CO (39/40, 97.5%) and spirometry (38/40, 95%) were the most commonly performed tests. Impulse oscillometry was reported as an additional test by 2/40 (5%) and fractional exhaled nitric oxide by 1/40, (2.5%). The frequency of post‐HSCT PFTs are summarized in Table 2. Pulmonary function tests were most commonly performed every 3 months (14/40, 35%) or 6 months (12/30, 30%) in the first year post‐HSCT, every 6 months (13/40, 32.5%) or 12 months (13/40, 32.5%) in the second year post‐HSCT, and every 12 months (21/40, 52.5%) thereafter. There were no significant differences between the responses of pulmonologists and HSCT physicians for any of the three time points. More than half of respondents (22/40, 55%) reported there was a set threshold for a significant drop in pulmonary function, and there was no significant difference in the responses of pulmonologists and HSCT physicians regarding this (44.0% vs. 73.3%, *p* = .095). In patients with known pulmonary complications most centers performed PFTs every 3 months (21/40, 52.5%) and for patients with known GVHD, PFTs were also performed every 3 months (18/40, 45%). In all centers, PFTs were interpreted by pediatric pulmonologists, however 3/40 (7.5%) respondents reported that adult pulmonologists sometimes interpreted tests. In addition to PFTs, the most commonly performed tests included a six‐minute walk test (6MWT, 21/40, 52.5%). The tests used in patients under 5 years of age are summarized in Table 3. Most reported using clinical symptoms and signs to assess such patients (34/40, 85%). Six respondents (15%) reported using conventional PFTs in this age group, one respondent (2.5%) used a 6MWT, and one (2.5%) used oxygen saturations.

**TABLE 2:** Frequency (%) of responses regarding pulmonary function tests after HSCT

Frequency	First year post‐HSCT	Second year post‐HSCT	After 2 years post‐HSCT	In patients with known late pulmonary complication	In patients with known GVHD
Monthly	0	0	0	7.5	7.5
Every 2 months	0	0	0	2.5	0
Every 3 months	35	2.5	0	52.5	45
Every 4 months	2.5	0	0	5	2.5
Every 6 months	30	32.5	2.5	5	7.5
Every 12 months	15	32.5	52.5	N/A*	N/A*
No routine screening, as clinically indicated	2.5	17.5	22.5	22.5	22.5
Unsure	15	15	22.5	5	15

*N/A as this was not an option for a response in the survey

**TABLE 3:** Summary of Tests used in patients 5 years and under

Test	Number of respondents (%)
Clinical symptoms and signs	34 (85)
MBW ‐ routinely scheduled	1 (2.5)
MBW ‐ as clinically indicated	0 (0)
Computed tomography ‐ Routinely scheduled	4 (10)
Computed tomography ‐ As clinically indicated	21 (52.5)
Impulse oscillometry ‐ Routinely scheduled	1 (2.5)
Impulse oscillometry ‐ As clinically indicated	2 (5)
Unsure	2 (5)
Other	
*Conventional PFT*	6 (15)
*6MWT*	1 (2.5)
*Oxygen saturations*	1 (2.5)

### 3.4 Imaging

The majority of respondents reported chest computed tomography (CT) scans were performed pre‐HSCT (21/40, 52.5%), however most reported they were not routinely repeated post‐HSCT (26/40, 65%).

### 3.5 Access to pediatric pulmonology

All 15 HSCT physician respondents reported working in a center with access to pediatric pulmonology. There was a relatively even distribution between respondents reporting no routine referral to pediatric pulmonology (21/40, 52.5%) and a routine referral (19/40, 47.5%). Again, there was an even distribution of whether centers had a defined pulmonologist for this patient group; 21/40 (52.5%) had a defined clinician, and 19/40 (47.5%) did not. There was no significant relationship between presence of a defined pulmonologist and whether centers had a protocol for monitoring for complications and reported adherence to the protocol.

### 3.6 Invasive diagnostic testing

All respondents worked in centers with access to bronchoscopy and all had been involved in a patient who had undergone bronchoalveolar lavage. Nearly all respondents had cared for a patient who had a lung biopsy (38/40, 95%), and in most cases this was looking for both infection and non‐infectious pathology (34/40, 85%). A variety of biopsy techniques were used (see Table 4) with thoracoscopic being the most common (29/40, 72.5%).

**TABLE 4:** Biopsy Method

Type of biopsy	Number of respondents (%)
Open surgical biopsy	23 (57.5)
Thoracoscopic biopsy	29 (72.5)
Transbronchial biopsy	10 (25)
Endobronchial ultrasound guided biopsy	3 (7.5)
Cryotherapy	0 (0)
Interventional radiology guided biopsy	8 (20)
Not applicable	2 (5)

## 4 DISCUSSION

The survey illustrates variation in practices for monitoring pulmonary health of pediatric HSCT survivors. We suspect that this is due to a lack of standardized guidelines, which reflects a paucity of data about which approaches are optimal. It may also reflect differences in access to diagnostic resources.^30^ Our findings highlight several priority areas for future research and quality improvement initiatives such as clinical guidelines to address when and how to monitor this high‐risk population.

International guidelines are consistent in recommending screening for pulmonary complications of HSCT.^1^, ^2^, ^27^ A concerning result of the current survey is 20% of respondents reporting no formal institutional protocol for monitoring pulmonary complications of pediatric HSCT. Given the increasing number of HSCT survivors over time, having a monitoring protocol in place helps ensure that patients are screened systematically and equitably. In addition, 25% of respondents reported “average” or “poor adherence” with the defined protocol at their center. There are limitations to this assessment as it was self‐reported, rather than a formal assessment of adherence. However, it raises concerns regarding whether patients are monitored appropriately even when protocols exist.

The survey also illustrates variations in practice regarding which PFTs are used for screening and the frequency of testing. This is unsurprising given the variation in international guidelines. In the current study, most respondents reported using spirometry and D_L_CO; 60% also used plethysmography. Small numbers of respondents used additional tests such as MBW and impulse oscillometry. Current guidelines focus on D_L_CO and spirometry. Spirometry is an insensitive test to assess airflow limitation at the peripheral airways, the site at which BO develops.^36^ Data suggests that MBW with calculation of the lung clearance index is more sensitive for detecting early changes of pulmonary GVHD,^28^, ^29^, ^36^ but several barriers exist in the widespread use of this test, including time constraints and lack of clinical experience among many providers. An advantage of both MBW and impulse oscillometry is the ability to use them in the preschool‐age population. Data from this study also highlights the variation in the frequency of testing post‐HSCT, which again may reflect conflicting existing guidelines. An additional issue is the absence of established test parameters that indicate disease in this population. In the current survey only 55% of respondents reported a set threshold for a significant drop in pulmonary function, and none of the international guidelines provide advice in this area.

A recent publication regarding health surveillance in HSCT survivors highlighted the need for a multidisciplinary approach.^37^ While there are no clear guidelines for the role of pulmonologists in HSCT survivor follow‐up, there are data regarding their current involvement. A survey of North American HSCT center directors demonstrated that 71% of centers without a dedicated follow up clinic had an identified pulmonologist they referred patients to, and in centers with a dedicated clinic, a pulmonologist was involved in 84.4% of those clinics.^38^ In the current survey, all respondents had access to pediatric pulmonologists, however only 52.5% reported there was an identified pulmonologist for referrals and 52.5% reported routine referral of patients to pediatric pulmonology. A potential explanation for the lower rates in this current survey is differences in practice between adult and pediatric HSCT centers. Follow‐up of children who have undergone HSCT is complex and subspecialty expertise is needed. The ideal scenario would involve at least one dedicated pulmonologist at each center working closely together with HSCT physicians to deliver patient care and develop screening programs and research projects to answer clinically relevant questions.

There are some limitations to the current project. Firstly, use of a survey approach relies on respondents' answers to questions rather than a direct assessment of practices. It would be expensive and impractical to perform in‐person assessments of practice across centers on two continents. Our survey concluded just prior to the start of the SARS‐CoV‐2 pandemic, and thus our results reflect pre‐pandemic practices.

Because 34.8% of invited participants responded, some bias may exist. The response rate, however, is similar to another survey of HSCT center directors that reported a response rate of 38.5%.^38^ Our main objective was to determine the breadth of practice. While a higher response rate might have provided a clearer picture of which practices are most common, it likely would not have dramatically affected our findings that practice patterns are highly variable. Our response rate was higher in pediatric pulmonologists meaning the results are more likely to reflect the practices this group. In addition, because we collected anonymous responses, it is possible the responses were mainly received from a distinct geographical area in the USA or Australasia, meaning the results may not represent practice across the whole region. The decision to survey both HSCT physicians and pediatric pulmonologists at the same center, may have led to conflicting responses which we could not ascertain due to the anonymous nature of the survey. We chose to keep the responses of the survey de‐identifiable as this maximized the likelihood of response and this was more important than identifying conflicting practices within a center. We surveyed both professional groups as even within the same institution, practice can differ between specialities, and it was important to solicit a broad range of clinical practice.

Lastly, even though clinicians in three different countries were surveyed, we acknowledge this does not represent an exhaustive assessment of practice patterns worldwide. Specifically, we do not have data on practice in more resource‐poor countries. Despite these limitations, given the lack of any data in this area, a survey represented a pragmatic and efficient way to gain knowledge from a large number of centers. Our data show, even given the above limitations, that significant variability of practice exists.

Future work can build upon the findings of this project by developing a consensus guidelines for pediatric HSCT centres to follow. These could be developed by a multidisciplinary group, including but not limited to HSCT physicians, pediatric pulmonologists, nurses, respiratory function scientists/respiratory therapists, radiologists and most importantly HSCT survivors and family members of children who have undergone HSCT. These guidelines could be endorsed by HSCT and pulmonary societies and could be continuously refined as evidence regarding optimal surveillance is generated. In particular the development of guidelines targeted at the follow up of children who have undergone allogenic HSCT is a priority. Allogenic and autologous HSCT survivors are at risk of different pulmonary complications and likely require different surveillance protocols. Most children who undergo autologous HSCT will be followed by primary oncologists with screening for complications likely following guidelines for solid tumor survivors issued by the Children's Oncology Group.^39^ The allogenic HSCT population is particularly vulnerable due to the relative lack of clear guidance and the increased risk of pulmonary complications and hence are the priority for development of consensus guidelines.

## 5 CONCLUSION

This survey reveals variations in screening for pulmonary complications of HSCT among children. Uniform and effective screening among pediatric HSCT survivors may minimize the detrimental effects of late pulmonary complications and result in improved quality of life and survival. Further research is essential to determine the optimal screening protocol, including the role of new tests such as MBW. However, simultaneous to this research there is a clear and urgent need for uniform clinical guidelines in this area, with consideration given to feasibility of implementation as broadly as possible, especially as it relates to unequal distribution of resources. This guidance could be derived from a rigorous systematic review of existing literature, led by an international group of HSCT physicians and pediatric pulmonologists. Such a guideline would likely reduce variation in care and lead to improved clinical outcomes for pediatric HSCT survivors.

## CONFLICT OF INTEREST

The authors have no conflicts to disclose.

## AUTHOR CONTRIBUTIONS

*Conceptualization; data curation; formal analysis; project administration; writing ‐ original draft; writing‐review & editing*, S.S.; *Conceptualization; formal analysis; writing ‐ original draft; writing‐review & editing*, W.G.; *Conceptualization; writing‐review & editing*, M.A. and N.A.; *Conceptualization; methodology; writing‐review & editing*, D.L., E.F., and T.C.; *Methodology; writing‐review & editing*, A.S., S.S., and T.V.; *Supervision; writing‐review & editing*, K.C.; *Conceptualization; supervision; writing‐review & editing*, S.G.

## ETHICS STATEMENT

Institutional review board approval was obtained from the Royal Children's Hospital, Melbourne, Australia (HREC number 2019.230). Completion of the electronic survey was viewed as consent to participate. In particular the start of electronic survey contained the following phrase: “*By completing the survey we assume you give informed consent to use your anonymous responses in our analysis and publications*.”

## Data Availability

The data that support the findings of this study are available from the corresponding author upon reasonable request.
